# Biophysical Characterization of the Dimer and Tetramer Interface Interactions of the Human Cytosolic Malic Enzyme

**DOI:** 10.1371/journal.pone.0050143

**Published:** 2012-12-21

**Authors:** Sujithkumar Murugan, Hui-Chih Hung

**Affiliations:** 1 Department of Life Sciences, National Chung Hsing University, Taichung, Taiwan; 2 Institute of Genomics and Bioinformatics, National Chung Hsing University, Taichung, Taiwan; 3 Agricultural Biotechnology Center, National Chung Hsing University, Taichung, Taiwan; University of South Florida College of Medicine, United States of America

## Abstract

The cytosolic NADP^+^-dependent malic enzyme (c-NADP-ME) has a dimer-dimer quaternary structure in which the dimer interface associates more tightly than the tetramer interface. In this study, the urea-induced unfolding process of the c-NADP-ME interface mutants was monitored using fluorescence and circular dichroism spectroscopy, analytical ultracentrifugation and enzyme activities. Here, we demonstrate the differential protein stability between dimer and tetramer interface interactions of human c-NADP-ME. Our data clearly demonstrate that the protein stability of c-NADP-ME is affected predominantly by disruptions at the dimer interface rather than at the tetramer interface. First, during thermal stability experiments, the melting temperatures of the wild-type and tetramer interface mutants are 8–10°C higher than those of the dimer interface mutants. Second, during urea denaturation experiments, the thermodynamic parameters of the wild-type and tetramer interface mutants are almost identical. However, for the dimer interface mutants, the first transition of the urea unfolding curves shift towards a lower urea concentration, and the unfolding intermediate exist at a lower urea concentration. Third, for tetrameric WT c-NADP-ME, the enzyme is first dissociated from a tetramer to dimers before the 2 M urea treatment, and the dimers then dissociated into monomers before the 2.5 M urea treatment. With a dimeric tetramer interface mutant (H142A/D568A), the dimer completely dissociated into monomers after a 2.5 M urea treatment, while for a dimeric dimer interface mutant (H51A/D90A), the dimer completely dissociated into monomers after a 1.5 M urea treatment, indicating that the interactions of c-NADP-ME at the dimer interface are truly stronger than at the tetramer interface. Thus, this study provides a reasonable explanation for why malic enzymes need to assemble as a dimer of dimers.

## Introduction

Malic enzyme (ME) is a homotetrameric enzyme catalyzing a reversible oxidative decarboxylation of L-malate to yield pyruvate and CO_2_ with the reduction of NAD(P)^+^ to NAD(P)H. This reaction requires a divalent metal ion (Mg^2+^ or Mn^2+^) for catalysis [1]–[3]. Malic enzymes are found in a broad spectrum of living organisms that share conserved amino acid sequences and structural topology, and these shared characteristics reveal a crucial role for the biological functions of these enzymes [4], [5]. In mammals, malic enzymes have been divided into three isoforms according to their cofactor specificity and subcellular localization as follows: mitochondrial NAD^+^-dependent ME (m-NAD-ME, EC 1.1.1.39), mitochondrial NADP^+^- dependent ME (m-NADP-ME, EC 1.1.1.40), and cytosolic NADP^+^-dependent ME (c-NADP-ME, EC 1.1.1.40). m-NAD-ME is found in rapidly proliferating tissues, particularly tumor cells [6], [7]. m-NADP-ME is found in tissues with low division rates, such as heart, muscle and brain tissue [2]. c-NADP-ME is expressed in liver and adipose tissues [2] and generates the NADPH required for fatty acid biosynthesis. In humans, c-NADP-ME is expressed in most tissues except for red blood cells [8], [9].

c-NADP-ME plays an important role in lipogenesis by providing NADPH for the biosynthesis of long-chain fatty acids and steroids. Thus, c-NADP-ME together with acetyl-CoA carboxylase, fatty acid synthase, and glucose-6-phosphate dehydrogenase are classified as lipogenic enzymes [2], [10]–[13]. c-NADP-ME has been characterized as an ideal target for the development of new drugs to reduce lipid levels [14]. In lipogenic tissues, such as liver and adipose, more than 90% of the malic enzyme activity is present in the cytoplasmic fraction [15]. High c-NADP-ME activity has also been observed in certain human carcinoma cell lines [10], [16], likely reflecting altered energy metabolism levels in cancer cells. The liver and adipose activities of c-NADP-ME are induced by a high carbohydrate/low fat diet and are down-regulated by a high fat diet [17]–[20]. Indeed, higher levels of liver c-NADP-ME activity have been associated with obese mouse and rat models [21], [22]. In addition, c-NADP-ME may play a significant role in the liver's detoxification of xenobiotics [23].

**Figure 1:**
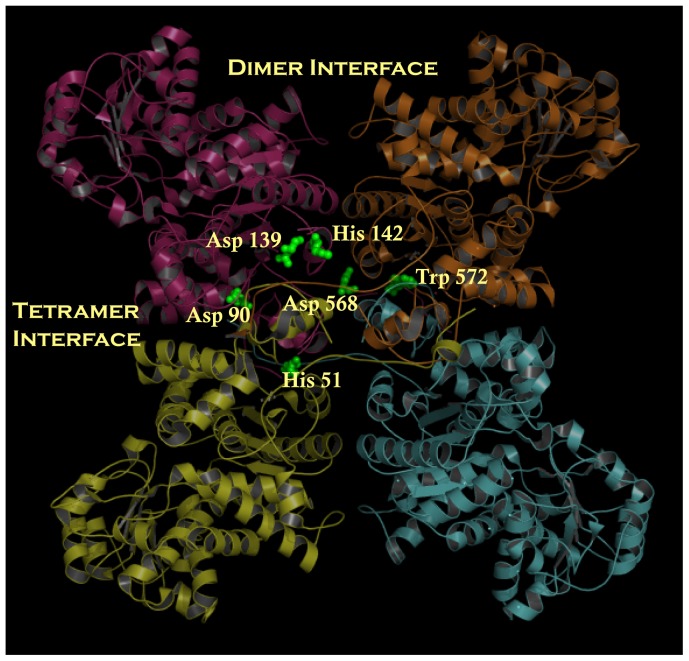
Dimer and tetramer interfaces of cytosolic malic enzyme. The dimer of dimers quaternary structure of cytosolic malic enzyme (PDB code: 1GQ2). The amino acid residues at the dimer interface, His 51, Asp 90, Asp 139, His 142, Asp 568 and Trp 572 in the dimer and tetramer interface are represented by ball-and-stick modeling. This figure was generated using PyMOL (DeLano Scientific LLC, San Carlos, CA).

Various crystal structures of malic enzymes in complex with substrate, metal ion, coenzyme, regulator, and inhibitor are available in the Protein Data Bank [4], [24]–[29]. The overall tertiary structures of these malic enzymes are similar, but there are still some differences that may be significant for catalysis and regulation. ME is composed of four identical monomers, each with its own active site. The tetramer of the human ME exists as a double dimer structure in which the dimer interface is more intimately contacted than the tetramer interface. The dimer interface is formed by subunits A and B or C and D, whereas the tetramer interaction consists of contacts between subunits A and D or B and C (Figure 1). Although the crystal structure of human c-NADP-ME is shown as a trimer (pdb code: 2AW5), it is believed to be a homotetramer [30] and similar to pigeon c-NADP-ME [26].

Here, we analyze the stability of the dimer and tetramer interface interactions of human c-NADP-ME. Our previous studies have demonstrated that introducing mutations at the dimer or tetramer interface result in the enzyme dissociating into left-and-right (AB or CD) or up-and-down (AD or BC) dimers [30]. In this study, the tetramer interface mutants H142A, H142A/D568A and W572A were assumed to be AB or CD dimers, and the dimer interface mutants H51A/D90A and H51A/D139A were assumed to be AD or BC dimers. Detailed kinetic data suggest that there are no differences in the enzymatic activity of c-NADP-ME when the enzyme dissociates into dimers and that AB or AD dimeric c-NADP-ME is as active as tetrameric c-NADP-ME [30].

In this study, the differences in the protein stability of AB or AD dimeric c-NADP-ME were examined using various biophysical methods. The urea-induced unfolding processes of these interface mutants were monitored using circular dichroism, fluorescence spectroscopy, analytical ultracentrifugation and enzyme activities. Here, we demonstrate the differential stability between the dimer and tetramer interface interactions of human c-NADP-ME. These data provide a reasonable explanation for why malic enzymes need to assemble as a dimer of dimers.

## Results

To discriminate between the differences in the biophysical properties of the tetramer and dimer interface mutants, W572A, H142A and H142A/D568A, which are assumed to be tetramer interface mutants (AB or CD dimer), and H51A/D90A and H51A/D139A, which are assumed to be dimer interface mutants (AD or BC dimer), two models were utilized [30]. In this study, protein stability was examined using thermal and urea-induced denaturation. In addition, because both the dimer and tetramer c-NADP-ME interface mutants are dimers, the quaternary structure changes of the WT and interface mutant enzymes were examined using analytical ultracentrifugation.

### Thermal denaturation of human c-NADP-ME WT and the interface mutants

We first examined the thermal stability of WT c-NADP-ME and interface mutant enzymes in the absence or presence of Mg^2+^, and the *T*_m_ values are shown in Table 1. The thermal stabilities of WT c-NADP-ME and the interface mutants with Mg^2+^ are apparently very similar to those without Mg^2+^. In addition, the *T*_m_ value of WT c-NADP-ME was approximately 60°C without Mg^2+^ and 62°C with Mg^2+^, indicating that the overall conformational stability of the enzyme is not highly dependent on Mg^2+^ ions.

**Table 1:** Thermal stability of WT human c-NADP-ME and the interface mutants.

	c-NADP-ME	*T*_m_ a without Mg^2+^ (°C)	*T*_m_ a with 4 mM Mg^2+^ (°C)
	WT	60	62
**Tetramer Interface Mutants**	W572A	60	61
	H142A/D568A	61	62
	H142A	63	64
**Dimer Interface Mutants**	H51A/D90A	52	53
	H51A/D139A	52	53

a These data were monitored using circular dichroism spectrometry.

For the tetramer interface mutants, the thermal stability of the enzyme was not changed, and the *T*_m_ values of these mutants were approximately 60∼64°C, which is similar to that of WT (Table 1). In contrast, for the dimer interface mutants, the thermal stabilities were significantly less stable than those of the WT and tetramer interface mutants, and the *T*_m_ values of these mutants were approximately 52∼53°C, which is 10°C lower than that of the WT and tetramer interface mutants (Table 1). These data indicate that the thermal stabilities of the interface mutants are significantly different. For the tetramer interface mutants (AB or CD dimer), their thermal stabilities were very similar to the tetrameric WT. However, for the dimer interface mutants (AD or BC dimer), their thermal stabilities were significantly reduced, suggesting that these two types of dimers have different thermal stabilities.

### Urea-induced denaturation of WT human c-NADP-ME and the interface mutants

**Figure 2:**
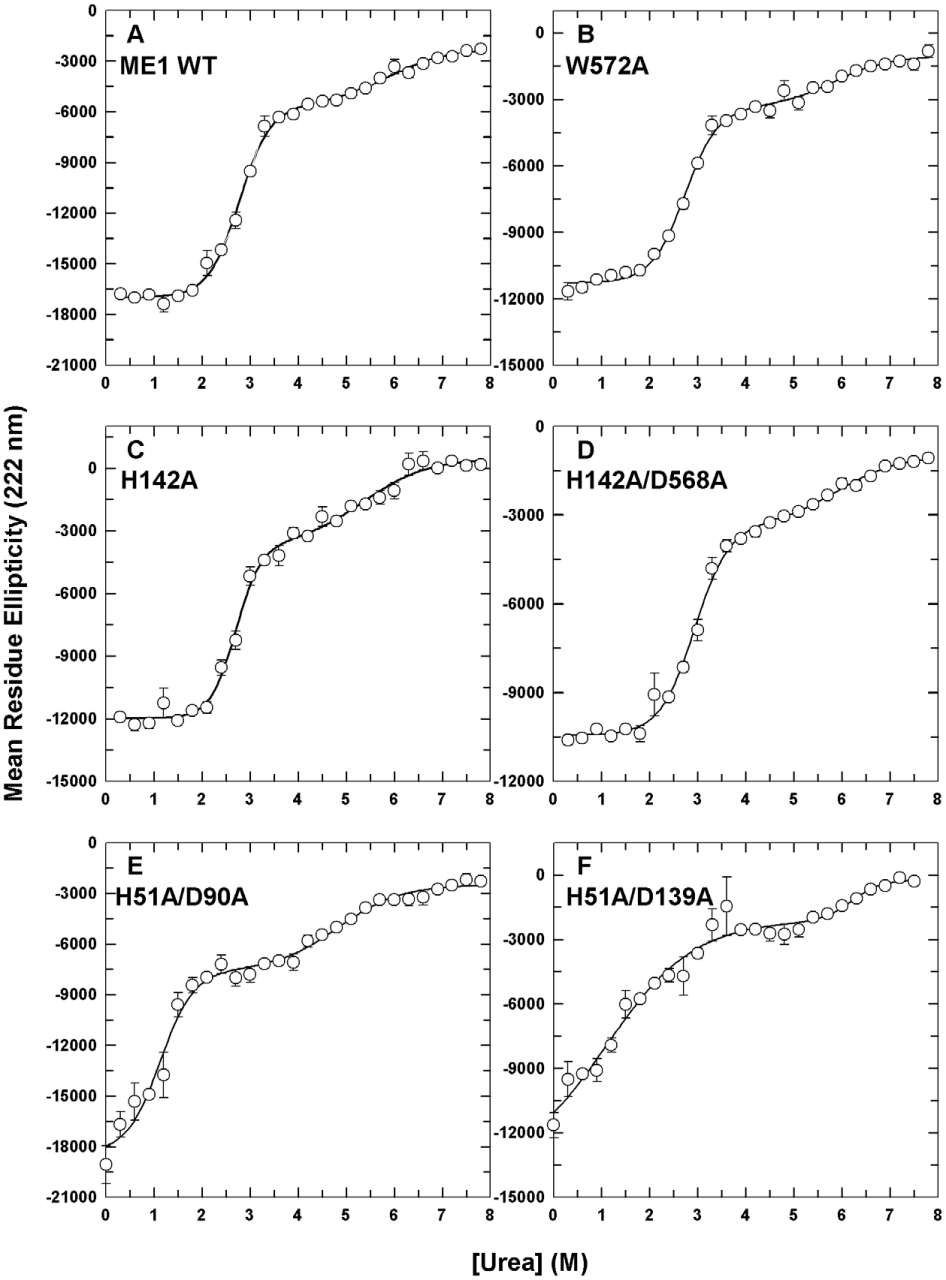
The urea-induced unfolding profiles of WT c-NADP-ME and interface mutants, as monitored using CD spectrometry. The enzymes were preincubated in various urea concentrations in 30 mM Tris-acetate (pH 7.4) at 25°C for 16 h. **A.** WT. **B.** W572A. **C.** H142A. **D.** H142/D568A. **E.** H51A/D90A. **F.** H51A/D139A. The experimental data are the mean residue ellipticity at 222 nm ((Θ)_222_) monitored by far-UV CD. All data were fitted using a three-state model. The fitting results and residues are displayed as a solid line with error bars.

We further examined the conformational stabilities of the WT c-NADP-ME and interface mutant enzymes using chemical denaturation (Figure 2) and determined their thermodynamic parameters (Table 2) by fitting them using a three-state unfolding model. The urea-induced denaturation of c-NADP-ME, represented by molar ellipticity curves (222 nm), demonstrated a biphasic behavior (Figure 2). For WT c-NADP-ME (Figure 2A), the urea concentrations of half-maximal denaturation, [Urea]_0.5_, for the first and second phases ([Urea]_0.5,N→I_ and [Urea]_0.5,I→U_, respectively) of the biphasic curve were 2.8 M and 5.8 M (Table 2).

**Table 2:** Thermodynamic parameters of WT human c-NADP-ME and the interface mutants during urea-induced denaturation.

	c-NADP-ME	[Urea]_0.5,N→I_ (M)^a^	[Urea]_0.5,I→U_ (M)^a^
	WT	2.8±0.4	5.8±3.5
**Tetramer Interface Mutants**	W572A	2.7±0.4	5.8±3.7
	H142A	2.7±0.6	5.3±3.2
	H142A/D568A	2.9±0.5	5.9±4.3
**Dimer Interface Mutants**	H51A/D90A	1.1±0.4	4.8±2.5
	H51A/D139A	1.1±0.8	6.2±5.1

a These data were derived from fitting the results in Figure 2 and were monitored using circular dichroism spectrometry.

For the tetramer interface mutants of c-NADP-ME, the urea denaturation curves were also demonstrated to be biphasic and similar to that of the WT (Figure 2, B–D). The [Urea]_0.5_ values of the W572A, H142A and H142A/D568A mutants were 2.7 M, 2.7 M and 2.9 M for the first phase and 5.8 M, 5.3 M and 5.9 M for the second phase, respectively (Table 2). These values were very similar to those of the WT (2.8 M and 5.8 M, respectively), suggesting that the conformational stabilities of the tetramer interface mutants (AB or CD dimer) are very similar to that of the WT.

For the dimer interface mutant H51A/D90A, although it displayed a biphasic denaturation curve (Figure 2E), its [Urea]_0.5_ values were smaller than those of the WT. Its [Urea]_0.5_ value was 1.1 M for the first phase and 4.8 M for the second phase (Table 2). The denaturation curves of the H51A/D139A mutant were also biphasic; however, a shoulder in its spectroscopic curves implied that an unstable intermediate existed at equilibrium (Figure 2F). Its [Urea]_0.5_ values were 1.1 M for the first phase and 6.2 M for the second phase (Table 2). These data indicate that the tetramer interface mutants demonstrate similar stabilities to WT. However, for the dimer interface mutants, the first transition of the denaturation significantly shifted towards a lower urea concentration, indicating less stability compared to the WT enzyme.

### Binding of ANS (8-anilino-l-naphthalene sulfonate) to the unfolding intermediate

**Figure 3:**
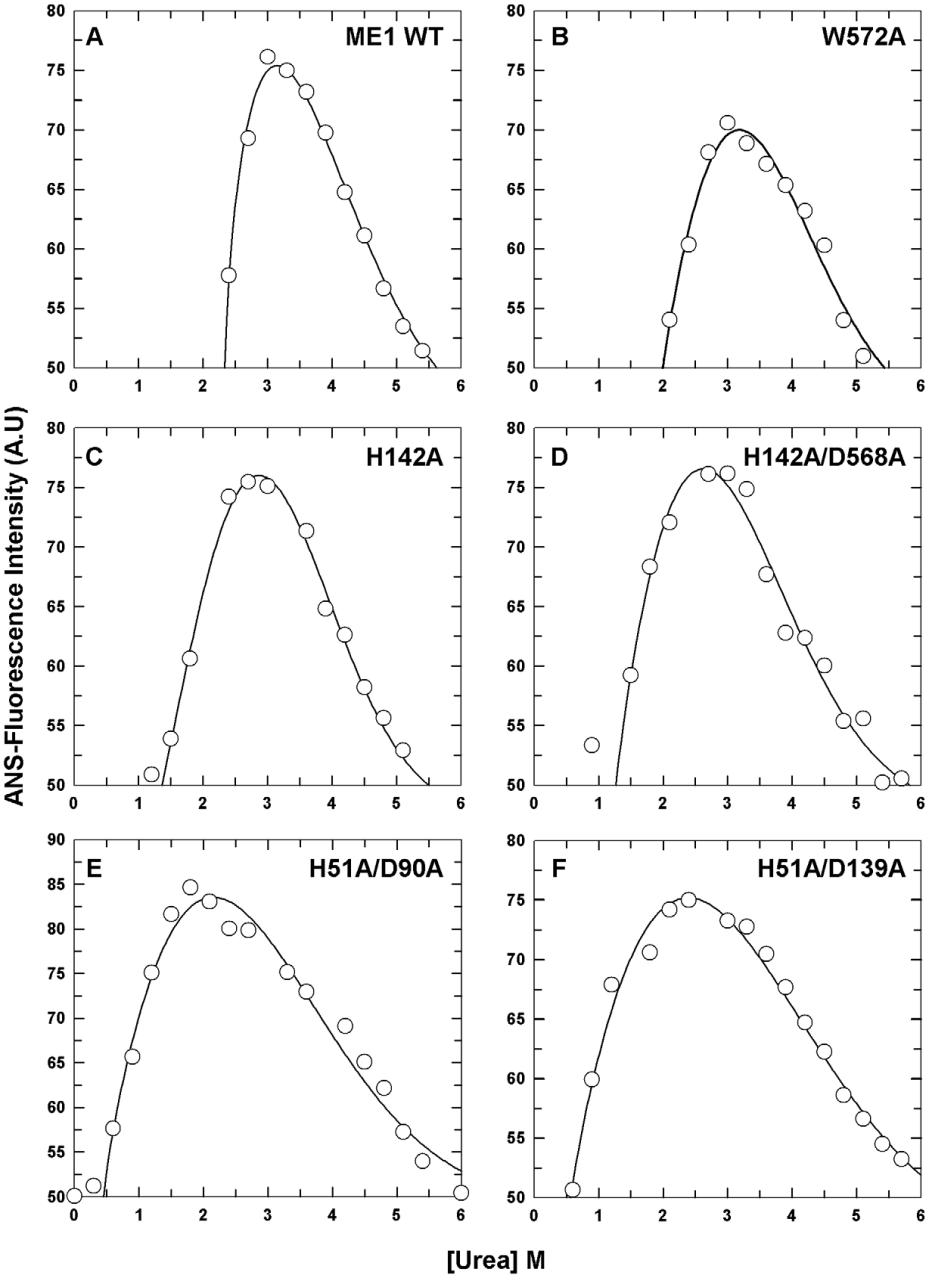
Intermediate states of WT human c-NADP-ME and the interface mutants, as detected using ANS-fluorescence. The enzymes were preincubated at various urea concentrations in 30 mM Tris-acetate (pH 7.4) at 25°C for 16 h and then incubated with 5 mM ANS for 10 min in the dark at 25°C. **A.** WT. **B.** W572A. **C.** H142A. **D.** H142/D568A. **E.** H51A/D90A. **F.** H51A/D139A. The experimental data were ANS-fluorescence monitored at an emission wavelength of 510 nm.

To gain further insight into the intermediate state, the binding of ANS to WT human c-NADP-ME and the interface mutants was studied as a function of the urea concentration. ANS is generally used as a reporter probe of hydrophobic surfaces on proteins. The denaturation of proteins results in the exposure of occluded hydrophobic sites, which can be visualized by the binding of ANS. It is well established that ANS binds with a high affinity to non-polar sites of proteins in the folded state and to hydrophobic intermediates, while it interacts very poorly with fully unfolded proteins [31]. Figure 3 demonstrates the changes in ANS-fluorescence for WT human c-NADP-ME and the interface mutants with increasing concentrations of urea. A bell-shaped curve with a single peak was observed, suggesting an unfolding intermediate was produced during the unfolding process. For the WT and tetramer interface mutants, the partially unfolded intermediate was observed at approximately 2.7–3.0 M urea (Table 3). For the dimer interface mutants, the unfolded intermediates of H51A/D90A and H51A/D139A were observed at approximately 1.8 and 2.4 M urea, respectively (Table 3). The dimer interface mutants demonstrated maximum ANS-fluorescence intensities at lower urea concentrations compared with the WT tetramer interfaces. However, these results coincided with the thermodynamic data derived from the urea-induced denaturation method that was monitored by CD (Table 2) because the maximal ANS-fluorescence was between the value of the [Urea]_0.5_ for the first and second phases ([Urea]_0.5,N→I_ and [Urea]_0.5,I→U_, respectively).

**Table 3:** Midpoints of ANS-fluorescence and half inactivation for WT human c-NADP-ME and the interface mutants during urea-induced denaturation.

	c-NADP-ME	C_M_ [M]^a^	[Urea]_0.5,N→U_ (M)^b^
	WT	3.0	2.0±0.4
**Tetramer Interface Mutants**	W572A	3.0	1.9±0.4
	H142A	2.7	1.7±0.3
	H142A/D568A	3.0	1.6±0.3
**Dimer Interface Mutants**	H51A/D90A	1.8	1.0±0.2
	H51A/D139A	2.4	0.9±0.2

a These data were derived from Figure 3 and were monitored using ANS-fluorescence.

b These data were derived from the fitting results in Figure 4 and were monitored using an activity assay.

### Residual enzymatic activity after urea-induced denaturation

When incubated with increasing urea concentrations, the residual enzymatic activities of WT human c-NADP-ME and the interface mutants displayed a two-state (native and unfolded) denaturation (Figure 4). This enzymatic activity was gradually lost with increasing [Urea]. For the WT and tetramer interface mutants, total inactivation of the enzyme occurred at 3 M urea, while for the dimer interface mutants, total inactivation occurred at approximately 2 M urea (Figure 4). In addition, for WT c-NADP-ME and the tetramer interface mutants, the urea concentrations of half-maximal denaturation, [Urea]_0.5,N→U_ of the monophasic curve, were approximately 1.6–2.0 M (Table 3); however, for the dimer interface mutants, this value was approximately 1 M, clearly indicating that enzymatically inactivating the dimer interface mutants requires less denaturant than the WT and tetramer interface mutants. Furthermore, the loss of the enzymatic activities of the WT and tetramer interface mutants was prior to protein structure perturbation because the [Urea]_0.5_ values of residual enzymatic activity (1.6–2.0 M, Table 3) were smaller than those of the first phase of denaturation, as monitored using CD (2.7–2.9 M, Table 2). In contrast, for the dimer interface mutants, the loss of enzymatic activity appeared to be concurrent with protein structure perturbation because the [Urea]_0.5_ values of residual enzymatic activity (0.9–1.0 M, Table 3) and the first phase of the denaturation, as monitored by CD, were almost identical (1.1 M, Table 2). In addition, the intermediate states of the tetramer and the dimer interface mutants were observed at approximately 3 M and 2 M urea (Table 3), respectively, indicating that the intermediate states of the enzymes were in an inactive form.

**Figure 4:**
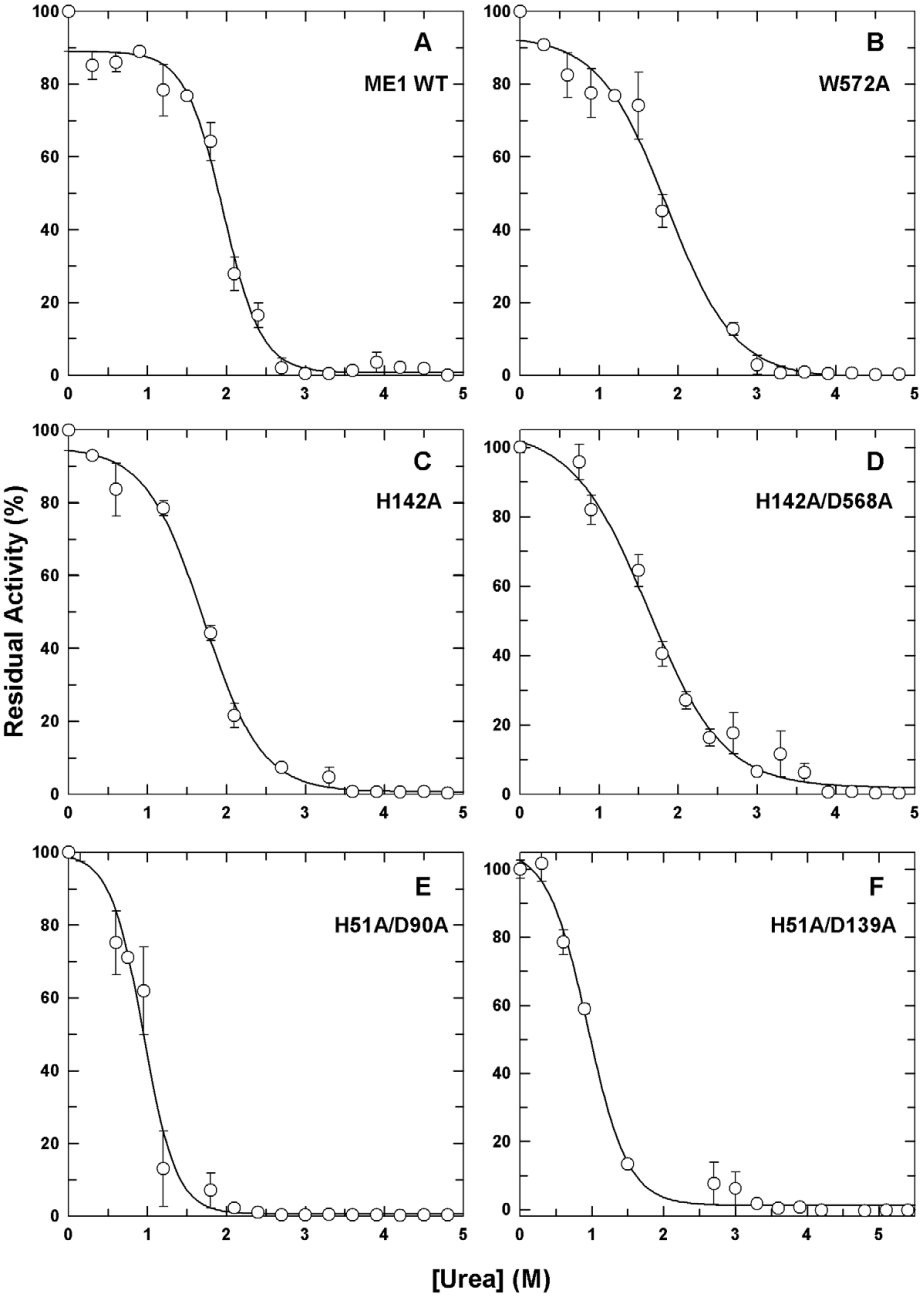
Urea-induced enzyme inactivation of WT human c-NADP-ME and the interface mutants. The enzymes were preincubated in various urea concentrations in 30 mM Tris-acetate (pH 7.4) at 25°C for 16 h and then monitored by following NADPH production at an absorbance of 340 nm. All data were fitted using a two-state model, and the fitting results and residues are displayed as a solid line with error bars.

### Sedimentation velocity measurements

**Figure 5:**
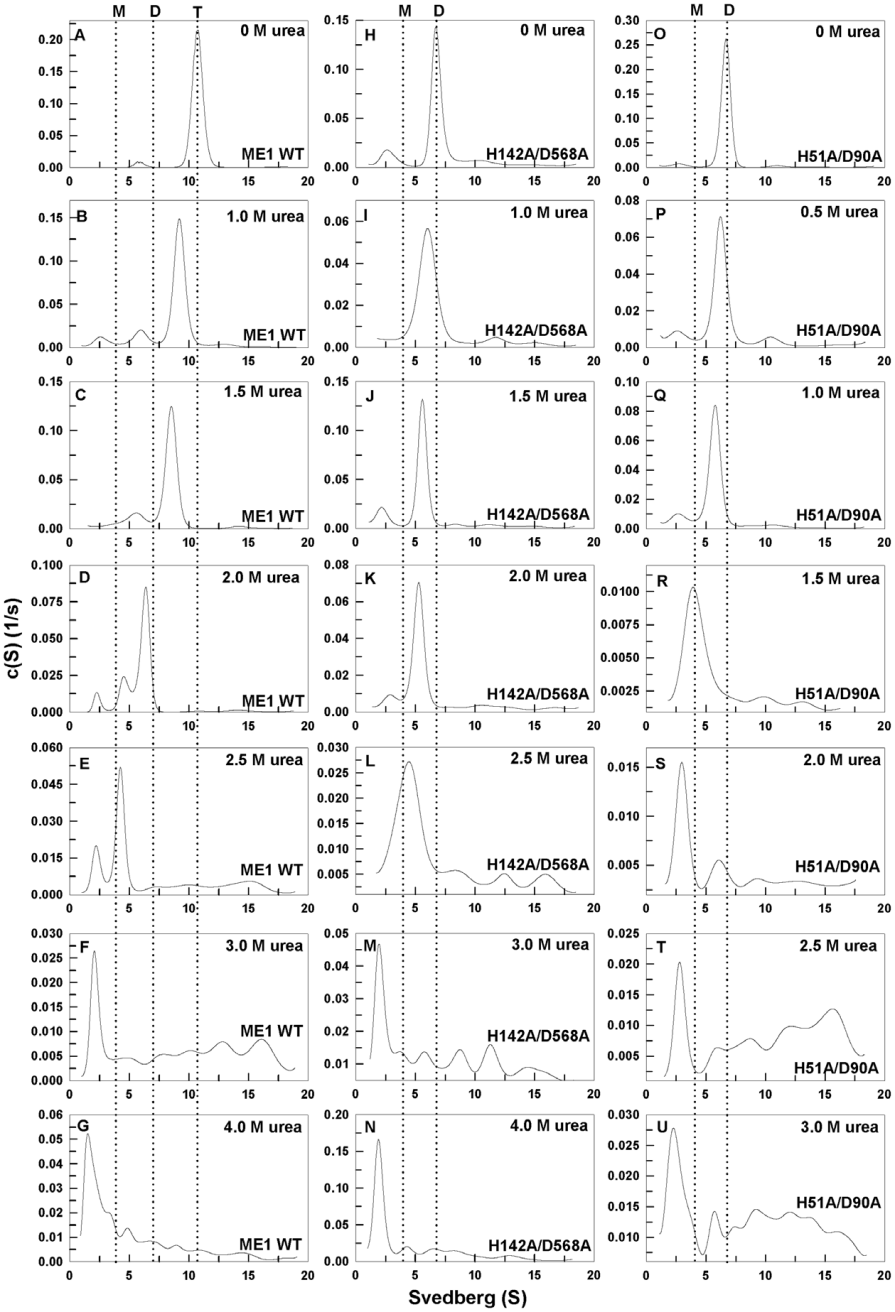
Continuous sedimentation coefficient distributions of WT c-NADP-ME and the interface mutants during urea denaturation. The enzymes were preincubated in various urea concentrations in 30 mM Tris-acetate (pH 7.4) at 25°C for 16 h and then run in an analytical ultracentrifuge at 20°C. **Panels A–G:** WT. **Panels H–N:** H142A/D568A. **Panels O–U:** H51A/D90A.

We examined the quaternary structural changes of WT human c-NADP-ME and the interface mutants under urea denaturation using analytical ultracentrifugation. Figure 5 shows the continuous sedimentation coefficient distribution of the WT and interface mutant enzymes during urea denaturation. The quaternary structure of WT human c-NADP-ME is a stable tetramer with a sedimentation coefficient of 10.7 S (Figure 5A), while the tetramer and dimer interface mutants of c-NADP-ME exist as stable dimers with a sedimentation coefficient of 7.2 S (Figures 5H and 5O, respectively). For tetrameric WT c-NADP-ME, the enzyme was first dissociated from tetramers to dimers before 2 M urea (Figure 5D), and the dimers then dissociated into monomers before 2.5 M urea (Figure 5E). These results also suggest that the first dissociation took place without a large degree of denaturation because the [Urea]_0.5_ for the first phase was 2.8 M (Table 2). For a dimeric form of the tetramer interface mutant H142A/D568A, the AB or CD dimer dissociated almost completely into monomers at 2.5 M urea (Figure 5L), while for the dimeric form of the dimer interface mutant H51A/D90A, the AD or BC dimer completely dissociated into monomers at 1.5 M urea (Figure 5R). The dissociation of these dimer interface mutants may have been due to a partial structural denaturation of the dimer. The H51A/D90A mutant dissociated into monomers at 1.5 M urea; however, the first transition of the denaturation occurred at 1.1 M (Table 2). These data clearly indicate that the interactions at the dimer interface are stronger than those at the tetramer interface. Therefore, the WT enzyme first dissociates into an AB or CD dimer, and the dimer then dissociates into monomers. The other dimer interface mutant enzymes also demonstrated similar dissociation patterns (Figure S1). The H142A and W572A mutants (AB or CD dimer) dissociated into monomers at 2.5 M urea, while the H51A/D139A mutants (AD or BC dimer) dissociated into monomers at 1.35 M urea.

## Discussion

Malic enzyme is a homotetramer of four identical subunits with the same structural folds. Based on the crystal structures of various isoforms of malic enzyme, the enzyme possesses a dimer-dimer quaternary structure in which the dimer interface is composed of relatively stronger interactions than the tetramer interface [24], [25]. Mutagenesis and biochemical studies have shown that the ionic interactions and hydrogen bonding stabilize the dimer and tetramer interfaces and hydrophobic interactions in the C-terminal domain are also an important factor for the tetramer interface organization [30]. Here, we used c-NADP-ME as a model to delineate the correlation between these interface interactions and the protein stability of the enzyme. This study clearly illustrates the biophysical differences in the structural stabilities between the dimer and tetramer interfaces of the enzyme. In addition, methods that differentiate the left-and-right dimer (AB or CD dimer) or up-and-down dimer (AD or BC dimer) have been successfully established.

### Dissociations of subunits at the dimer interface, but not at the tetramer interface, significantly affect the stability of c-NADP-ME

Our data clearly indicate that the protein stability of c-NADP-ME is predominantly affected by disruptions at the dimer interface rather than at the tetramer interface. The disruption of the dimer interface of c-NAD(P)-ME causes the enzyme to be less stable. The first piece of evidence supporting this hypothesis is the fact that, during thermal stability experiments, the melting temperature of the WT and tetramer interface mutant enzymes were 8–10°C higher than those of the dimer interface mutant enzymes (Table 1), truly reflecting that the dissociation of subunits at the dimer interface, but not at the tetramer interface, significantly affects the stability of the enzyme.

Second, during the urea denaturation experiments, the thermodynamic parameters of the WT and tetramer interface mutants were almost identical, and the unfolding intermediates existed at a similar urea concentration, suggesting that the c-NADP-ME tetramer and AB or CD dimers possess very similar structural stabilities. However, for the dimer interface mutants, the first transition of the urea unfolding curves shifted towards a lower urea concentration (Table 2), and the unfolding intermediate was stable at a lower urea concentration (Table 3), supporting the conclusion that the AB or CD dimer is more stable than the AD or BC dimer. Because the four subunits have identical structural folds, the different structural stabilities between the AB and AD dimers can be predominantly attributed to the different subunit-subunit interactions between the tetramer and dimer interfaces of the enzyme. Thus, the subunit-subunit interactions of c-NADP-ME at the dimer interface are truly stronger than at the tetramer interface.

The unfolding intermediates are inactive monomers. For the WT enzyme and the tetramer interface mutants, the unfolding intermediate (approximately 2.7–3 M urea, Table 3) occurred when the enzyme's activity was completely lost (approximately 3.0 M urea, Figure 4) and when the tetrameric WT and AB or CD dimers were monomers (2.5 M urea, Figure 5). For the dimer interface mutants, the dissociation from dimers to monomers (1.5 M urea, Figure 5) occurred before the formation of the unfolding intermediate (above 1.8–2.4 M urea, Table 3), and the unfolding intermediate also occurred when the enzyme's activity was completely lost (approximately 2.0 M urea, Figure 4). These data suggest that the dissociation of the enzyme and the loss of enzymatic activity are prior to the structural change of the enzyme and imply that a precise formation in the secondary and tertiary structures is important for the correct organization of the quaternary structure as well as for full enzymatic activity.

### Why does the non-cooperative and non-allosteric cytosolic NADP^+^-dependent malic enzyme need to form a tetramer?

Unlike m-NAD-ME, c-NADP-ME is a non-cooperative and non-allosteric enzyme. Therefore, it is clear that the formation of the c-NADP-ME tetramer is not for enzyme regulation. For this enzyme isoform, the AB or CD dimer is as stable as the tetramer in the secondary, tertiary and quaternary structures, which ensures that its enzymatic activity is also stable. Meanwhile, the AD or BC dimer is less stable than the WT in terms of its secondary, tertiary and quaternary structures, which affects its enzyme stability. During the folding process, both the AB and AD dimers may exist in equilibrium; however, the AB dimer may be more predominant than the AD dimer because the interactions between A and B are stronger than those between A and D. Because the AD or BC dimer is much less stable than WT, it is essential for the AD or BC dimer to form a tetramer. Therefore, even the AB or CD dimer displays enzymatic activities similar to that of the tetrameric enzyme and is apparently fully functional, a tetrameric structure of the enzyme is preferred for maximal stability. So why does this enzyme form a tetramer? This study has provided some information with regards to this question for this enzyme family.

## Materials and Methods

### Expression and purification of recombinant c-NADP-ME

c-NADP-ME was subcloned into the pET21b vector, which carries a C-terminal 6× His-tag sequence. This ampicillin-resistant vector was transformed into *Escherichia coli* BL21(DE3), and expression was controlled using an inducible T7 promoter system. The overexpressed enzyme was purified using a HIS-Select™ Nickel Affinity Gel column (Sigma). The lysate-Ni-NTA mixture was washed with buffer (10 mM imidazole, 500 mM sodium chloride, 2 mM *β*-mercaptoethanol, and 30 mM Tris-acetate (pH 7.4)) to remove contaminating proteins. The recombinant c-NADP-ME enzyme was recovered from the resin using elution buffer (250 mM imidazole, 500 mM sodium chloride, 2 mM β-mercaptoethanol, and 30 mM Tris-acetate buffer (pH 7.4)). The purified enzyme was dialyzed and concentrated using a centrifugal filter device (Amicon Ultra-15, Millipore, Billerica, MA) with a molecular mass cutoff of 30 kDa. Enzyme purity was verified by SDS-PAGE, and the protein concentrations were estimated using the Bradford method [32].

### Construction of c-NADP-ME interface mutants

Mutagenetic replacement was performed using the QuikChange™ kit (Stratagene, La Jolla, CA) to construct the mutated human c-NADP-ME plasmids. Purified human c-NADP-ME DNA was used as a template, and the specific primers with the desired codon and a high fidelity Pfu DNA polymerase were used in the PCR reaction. The lengths of the primers, including the desired mutation sites, were approximately 25- to 45-mer, which is considered necessary for the specific binding of the template DNA. After 16–18 temperature cycles, the mutated plasmids, including staggered nicks, were made. The PCR products were subsequently treated with *Dpn*I to digest the WT human c-NADP-ME templates. Finally, the nicked DNA of the desired mutations was transformed into *E. coli* strain XL-1, and the DNA sequences were verified using autosequencing.

### Thermal stability of c-NADP-ME

The thermal denaturation of WT human c-NADP-ME and the interface mutants was performed in Tris-acetate buffer (30 mM, pH 7.4) with or without 4 mM MgCl_2_. Circular dichrosim (CD) spectrometry was performed using a Jasco J-815 spectropolarimeter and a 0.1-cm quartz cuvette with a 1-nm slit bandwidth. The optical chamber of the CD spectrometer was deoxygenated with dry purified nitrogen (99.99%) for 45 min before use and kept under a nitrogen atmosphere during experiments. The thermal denaturation experiments were performed by increasing the temperature from 20 to 90°C. The temperature was increased 0.4°C/min, which was controlled using a circulating water bath, and the ellipticity at 222 nm of all samples was recorded to analyze the thermal stability of the proteins during the heating process.

### Monitoring the urea-induced denaturation of c-NADP-ME using circular dichroism spectrometry

The conformational stabilities of WT human c-NADP-ME and the interface mutants were further measured by equilibrium denaturation in the presence of urea (0–8 M). The WT and interface mutant enzymes were pre-incubated with various concentrations of urea in Tris-acetate buffer (30 mM, pH 7.4) at 25°C for 16 h to enable the unfolding reactions to reach equilibrium. Circular dichroism (CD) spectrometry was performed using a Jasco J-815 spectropolarimeter and 0.1-cm quartz cuvettes with a 1-nm slit bandwidth. The ellipticity (222 nm) of all samples was recorded to analyze the protein conformational changes during the urea unfolding process. The mean residue ellipticity (Θ) at 222 nm was calculated using the following equation:
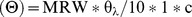
where MRW is the mean residue weight, θ_λ_ is the measured ellipticity in degrees at wavelength λ, l is the cuvette path length (0.1 cm), and c is the protein concentration (g/ml).

### Determination of surface hydrophobicity using ANS-fluorescence

ANS (8-anilino-l-naphthalene sulfonate) is an extrinsic fluorescent probe that binds to the hydrophobic patches present on a protein's surface. Thus, protein-bound ANS-fluorescence represents the surface hydrophobic properties of a protein molecule. The change in surface hydrophobicity of WT human c-NADP-ME and the interface mutants during urea-induced denaturation was further measured using ANS-fluorescence.

The enzyme was denatured using various concentrations of urea for 16 h at 25°C. ANS was then added to the protein sample before measuring the fluorescence. The ANS-fluorescence of the protein was measured using a Hitachi F-4500 FL luminescence spectrometer at 25°C, and all spectra were corrected for the buffer absorption. The excitation and emission slits were kept at 5 nm, and the excitation and emission wavelengths were set at 370 nm and 510 nm, respectively, to monitor the change in surface hydrophobicity of the enzyme during the urea-induced unfolding process.

### Enzymatic activity measurements of the c-NADP-ME in the presence of urea

The malic enzyme reaction was assayed in a reaction buffer including saturating concentrations of L-malate, NADP^+^ and MgCl_2_ in 50 mM Tris-HCl (pH 7.4) in a total volume of 1 ml at 30°C. Before measuring the residual enzymatic activity, the enzyme was incubated with various concentrations of urea for 16 h at 25°C. Changes in absorbance at 340 nm (ΔA_340_) were continuously recorded using a UV/VIS Spectrophotometer Lambda 25 (Perkin Elmer, USA).

### Analysis of the urea-induced denaturation curve

Analyses of the unfolding curves of the denaturation process were performed as described by Pace [33] and assumed a two-state or three-state unfolding mechanism. For a two-state (native and unfolded state) model, the 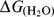 and *m* values were estimated by fitting the data to the following equation:
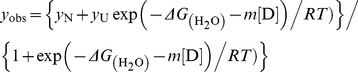
where *y*_obs_ denotes the observed signal change and *y*_N_ and *y*_U_ represent the signals for the folded and unfolded states, respectively. 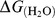 denotes the intrinsic free energy change in the absence of the denaturant, and *m* represents the dependence of the Δ*G* on the denaturant. [D] denotes the denaturant concentration, *T* is the absolute temperature in Kelvin, and *R* is the gas constant.

The denaturation curve was also analyzed using a three-state (native, intermediate and unfolded state) model. The 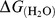 and *m* values at each step were estimated by fitting the overall data to the following equation:
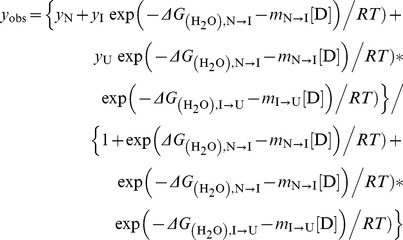
where *y*_I_ represent the signals for the intermediate states, 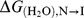 and 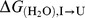 denote the intrinsic free energy changes for the N→I and I→U processes, and *m*_N→I_ and *m*_I→U_ are the *m* values for the corresponding processes, respectively. The concentration of urea for the half-denaturation of protein, [Urea]_0.5_, was estimated by dividing the Δ*G* by *m*.

### Quaternary structure analyses using analytical ultracentrifugation

To examine the quaternary structural changes of WT c-NADP-ME and the interface mutants during urea denaturation, before ultracentrifugation, the protein sample (0.3 mg/ml) was preincubated with various concentrations of urea (0∼4 M) at 25°C for 16 h. The sedimentation velocity experiments were performed using a Beckman Optima XL-A analytical ultracentrifuge. Samples (380 µl) and buffer (400 µl) solutions were separately loaded into the double sector centerpiece and built up in a Beckman An-50 Ti rotor. Experiments were performed at 20°C and a rotor speed of 42,000 rpm. Protein samples were monitored by UV absorbance at 280 nm in a continuous mode with a time interval of 480 seconds and a step size of 0.002 cm. Multiple scans at different time points were fitted to a continuous size distribution model using the program SEDFIT [34]. All size distributions were solved at a confidence level of p = 0.95, a best fit average anhydrous frictional ratio (*f/f_0_*), and a resolution N of 200 sedimentation coefficients between 0.1 and 20.0 S.

## Supporting Information

Figure S1 **Continuous sedimentation coefficient distributions of WT c-NADP-ME and the interface mutants during urea denaturation.** The enzymes were preincubated in various urea concentrations in 30 mM Tris-acetate (pH 7.4) at 25°C for 16 h and then run in an analytical ultracentrifuge at 20°C. **Panels A–G:** H142A. **Panels H–N:** W572A. **Panels O–U:** H51A/D139A.
(TIF)
